# Epigenetic Factors that Control Pericentric Heterochromatin Organization in Mammals

**DOI:** 10.3390/genes11060595

**Published:** 2020-05-28

**Authors:** Salvatore Fioriniello, Domenico Marano, Francesca Fiorillo, Maurizio D’Esposito, Floriana Della Ragione

**Affiliations:** Institute of Genetics and Biophysics ‘A. Buzzati-Traverso’, CNR, 80131 Naples, Italy; salvatore.fioriniello@igb.cnr.it (S.F.); domenico.marano@igb.cnr.it (D.M.); francesca.fiorillo@igb.cnr.it (F.F.)

**Keywords:** Pericentric heterochromatin, DNA methylation, repressive compartments, satellite DNA, MeCP2, ATRX, HP1, non-coding RNAs

## Abstract

Pericentric heterochromatin (PCH) is a particular form of constitutive heterochromatin that is localized to both sides of centromeres and that forms silent compartments enriched in repressive marks. These genomic regions contain species-specific repetitive satellite DNA that differs in terms of nucleotide sequences and repeat lengths. In spite of this sequence diversity, PCH is involved in many biological phenomena that are conserved among species, including centromere function, the preservation of genome integrity, the suppression of spurious recombination during meiosis, and the organization of genomic silent compartments in the nucleus. PCH organization and maintenance of its repressive state is tightly regulated by a plethora of factors, including enzymes (e.g., DNA methyltransferases, histone deacetylases, and histone methyltransferases), DNA and histone methylation binding factors (e.g., MECP2 and HP1), chromatin remodeling proteins (e.g., ATRX and DAXX), and non-coding RNAs. This evidence helps us to understand how PCH organization is crucial for genome integrity. It then follows that alterations to the molecular signature of PCH might contribute to the onset of many genetic pathologies and to cancer progression. Here, we describe the most recent updates on the molecular mechanisms known to underlie PCH organization and function.

## 1. Introduction 

Pericentric heterochromatin (PCH) is a fraction of the heterochromatin that is located on both sides of centromeres and that is usually replicated late during S phase [1], although exceptions have been reported for some organisms [2,3]. PCH is strongly enriched in repressive epigenetic marks, and is considered a paradigmatic example of constitutive heterochromatin in mammals [4]. PCH is crucial for preserving the integrity of the genome, repressing spurious transposition, and promoting correct chromosomal segregation [5,6,7]. 

At the molecular level, a repressive heterochromatic environment is established at pericentric repeats in both humans and mice, although the composition of PCH in these two species shows many differences. These genomic regions contain species-specific repetitive satellite DNA that differs in terms of nucleotide sequences, sizes of repeats, and lengths of arrays [8,9]. This demonstrates that the formation of heterochromatin is independent of its DNA sequence. 

**Figure 1:**
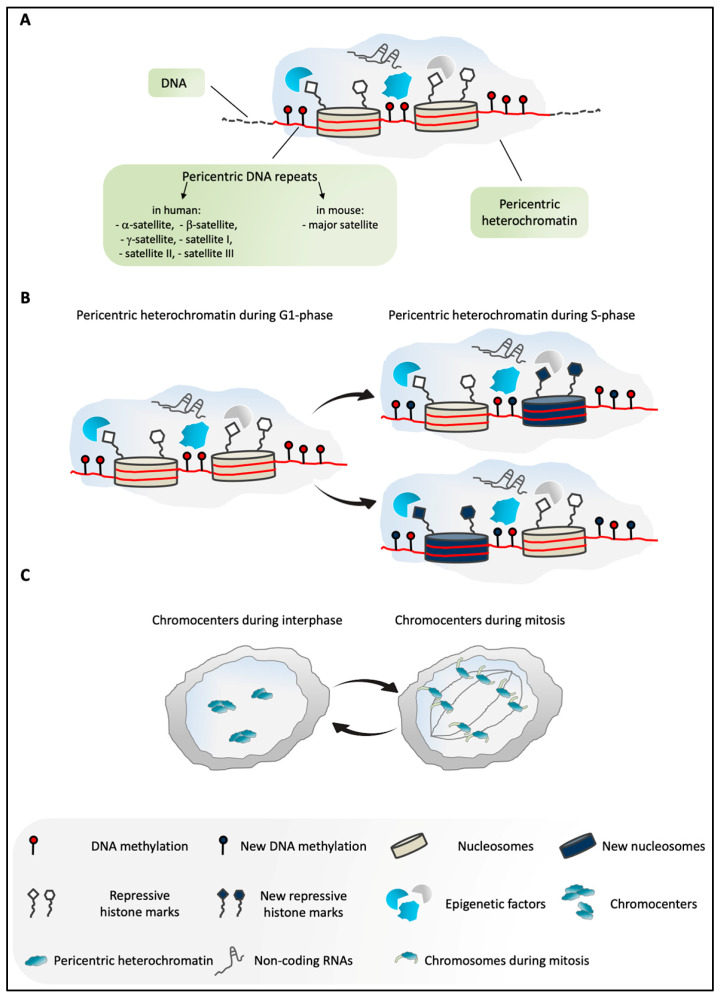
(**A**) Overview of pericentric heterochromatin (PCH) in mammals. PCH is constituted by highly methylated pericentric DNA repeats (α-, β-, and γ-satellites, satellites I, II, III in humans; major satellite in mice) [9,16]. It is enriched in several epigenetic factors, non-coding RNAs, and repressive histone modifications [4,13]. (**B**) Schematic representation of PCH in G1 and S phases of the cell cycle in mammals. According to the current model, during DNA replication, PCH is assembled through the incorporation of both old and newly synthesized histones. Similarly, epigenetic marks are enriched at PCH, including DNA methylation and repressive histone modifications, and these are inherited from the parental structure and/or established ex novo by different epigenetic factors. These processes ensure faithful maintenance of the PCH structure and its repressive environment during the cell cycle [4,13]. (**C**) In murine cells in interphase, PCH of different chromosomes is organized in highly compacted structures, termed chromocenters. During mitosis, the dissociation of the chromocenters into individual chromosomes takes place [4,16].

The repetitive DNA that makes up murine PCH is defined as major satellite (MajSat) DNA (Figure 1A), and this consists of several Mbp arrays of 234 bp-long repeats. These sequences are A/T rich, they represent ~5% of the genome, and they are located on all chromosomes that are acrocentric in mice [4].

Unlike the relatively simple composition and the uniform chromosomal distribution of murine repetitive PCH DNA, human pericentric regions contain different classes of repetitive DNA, including β-satellite and γ-satellite, and satellites I, II, and III (Figure 1A), which show diverse nucleotide compositions and lengths, and are differentially distributed on the chromosomes [9]. Moreover, α-satellite is also present in human PCH, although with a “noncanonical” organization, as it is interspersed with simple repeat DNA sequences and transposable elements [9,10].

A distinct combination of heterochromatic marks and the presence of some particular proteins characterize PCH in these two species. However, the common features of mouse and human PCH, which include DNA methylation, hypoacetylation of histones, enrichment of repressive histone marks, and the particular structural components [11,12] (Figure 1A), underline the importance of the common global chromatin organization that makes PCH an epigenetically defined entity. The intricate organization of PCH is finely orchestrated by the combined actions of several molecules, including structural proteins, chromatin remodelers, enzymes, and non-coding RNAs (ncRNAs), which all contribute to the higher-order PCH structure. Each of these factors plays a specialized role in the establishment and maintenance of the molecular signature of the pericentric regions.

The global organization of PCH and its repressive environment are preserved during the cell cycle, and are faithfully inherited [4,13]. According to the current model, during S phase, PCH is replicated, on the one hand, by inclusion of the histones that derive from the parental structure, together with their relative post-translational modifications, and, on the other hand, by incorporation of the newly synthesized histones and the establishment of the molecular signature of PCH ex novo by different epigenetic factors [4,13] (Figure 1B).

The spatial organization of PCH in the nucleus has been extensively studied in mouse cells. In interphase nuclei of several species, the PCH of different chromosomes aggregates to form distinct structures that are defined as chromocenters (Figure 1C), which are clearly visible using 4’,6-diamidino-2-phenylindole (DAPI) staining [11,14], due to their enrichment in the A/T nucleotides [11,15]. During mitosis, these chromocenters undergo temporary disaggregation (Figure 1C), and the DAPI spots highlight the PCH of the individual chromosomes [4,16].

The sizes and the numbers of chromocenters is cell-type specific, and is subject to changes during differentiation. These changes generally consist of clustering of the chromocenters, which thus increase in size and decrease in number [15,17,18,19,20]. A number of epigenetic factors contribute to this chromocenter clustering during both myogenic and neural differentiation, such as Methyl-CpG binding protein 2 (MeCP2) [18,19] and Alpha-thalassemia/mental retardation syndrome X-linked protein (ATRX) [20,21]. The biological significance of this particular organization of PCH remains elusive to date. However, several studies support the hypothesis that these heterochromatic structures represent repressive nuclear compartments in which silencing factors are concentrated [4,22,23]. 

Despite its heterochromatic nature, satellite DNA included in PCH can be actively transcribed to generate satellite ncRNAs that can then participate in the organization of chromatin structure in cis. Aberrant expression of these transcripts has been associated with pathological conditions, such as stress and cancers [9].

In this review, we provide an updated overview of the structure and function of PCH in mammals, under both physiological and pathological conditions. Here, we describe the molecular composition of PCH, with special attention paid to the different forms of DNA methylation, specific histone modifications, and the factors responsible for reading epigenetic marks and enzymatic components involved in the establishment and maintenance of PCH. In addition, the role of ncRNAs in these processes is described. Moreover, we summarize the main functional roles that have at present been linked to PCH.

## 2. Epigenetic Landscape at Pericentric Heterochromatin 

The most relevant epigenetic features of PCH are the typical histone modifications and methylation of pericentric DNA repeats. PCH is enriched in particular post-translational modifications of histone tails, such as dimethylation and trimethylation of lysine 9 of histone H3 (H3K9me2 and H3K9me3, respectively) [6,24], trimethylation of lysine 20 of histone H4 (H4K20me3) [24,25], and broad histone hypoacetylation [26]. H3K9me3 and H4K20me3 have been identified as central hallmarks of PCH in mammals [25,27]. In these heterochromatic regions, H3K9me3 can act as a docking site for specific factors, for the subsequent establishment of additional histone marks, such as H4K20me3, or to promote DNA methylation [25,28,29].

Following DNA replication, PCH shows enrichment in monomethylated H3K9 and H4K20 (H3K9me1 and H4K20me1, respectively), which are histone marks that are required for the preservation of chromocenter organization in DAPI-dense foci and for the subsequent establishment of H3K9me3 and H4K20me3 [30,31,32,33]. 

Two other histone modifications are enriched at PCH: H3K27me1 [31] and H3K64me3 [34]. The biological role of H3K27me1 for pericentric regions is still debated, whereas the function of H3K64me3 has been correlated with the stabilization of DNA–histone interactions, and the recruitment of histone and DNA methyltransferases (HMTs and DNMTs, respectively). These histone marks might ensure the appropriate epigenetic state of PCH [34].

The deposition of H3K64me3 on PCH is strictly dependent on the presence of H3K9me3, and appears not to require DNA methylation, H4K20me3, and heterochromatin protein 1 (HP1). Conversely, ablation of H3K64me3 impairs the recruitment of these PCH marks, which highlights the role of H3K64me3 in the reinforcement of the feedback loops during heterochromatinization of pericentric regions [35]. 

The maintenance of correct histone modification patterns in PCH, which include histone deacetylation, is crucial for the preservation of PCH molecular organization. Inhibition of histone deacetylases leads to altered distributions of chromocenters and HP1s, which move to the nuclear periphery and the nucleoplasm, respectively [7].

**Figure 2:**
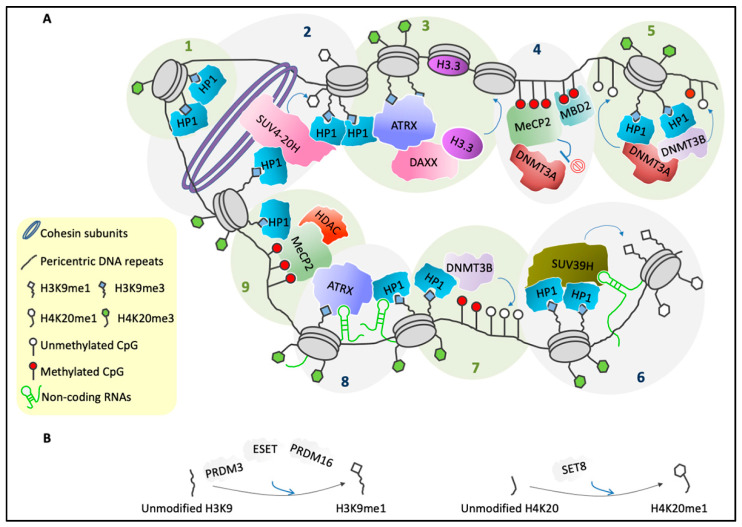
Several factors are involved in PCH organization. (**A**) Schematic representation of the general molecular structure of mammalian PCH during interphase. Step 1: HP1s bind to H3K9me3 and can self-interact [44]. Step 2: HP1s recruit the SUV4-20H enzymes, which can convert H4K20me1 into H4K20me3. Moreover, SUV4-20H binds cohesin subunits as well as HP1s, which reinforces chromatin compaction [25,45]. Step 3: HP1s and H3K9me3 provide a binding platform for ATRX [46,47], which in complex with DAXX mediates deposition of H3.3 [48]. Step 4: MeCP2 and MBD2 can form heterodimers [49], and they bind methylated CpGs [50]. Moreover, MeCP2 recruits DNMT3A and maintains it in a reversible inactive state [51]. Step 5: HP1s recruit DNMT3A and DNMT3B [16,40], which catalyze methylation of CpGs [52] and can form heterodimers [40]. Step 6: HP1–HP1 dimers recruit the SUV39H enzymes [39] that can trimethylate H3K9me1 on adjacent nucleosomes [16,31]. SUV39H binding to PCH is stabilized by an RNA component [53]. Trimethylation of H4K20me1 requires pre-existing H3K9me3 and HP1s [25]. Step 7: HP1s recruit DNMT3B, which then methylates CpGs [40,52]. Step 8: Accumulation of HP1 [54] and ATRX [20] at PCH also requires an RNA component. Step 9: ATRX binds MeCP2 [55,56], which then recruits a complex that has histone deacetylase activity [14]. Furthermore, MeCP2 interacts with HP1s [57]. (**B**) PRDM3, ESET, and PRDM16 promote the conversion of unmethylated H3K9 into H3K9me1 [32] (left). SET8 catalyzes the monomethylation of H4K20 tails [58,59] (right).

Post-translational modifications of histone tails at PCH are established through combined actions of a plethora of enzymes. Trimethylation of H3K9 is mediated by the HMT suppressor of variegation 3-9 homolog 1 (SUV39H1) [31,36]. SUV39H1 includes a suppressor of variegation, enhancer of zeste, and trithorax (SET) domain that has HMT activity, and a chromodomain that can specifically bind H3K9me2 and H3K9me3 and therefore targets SUV39H1 to PCH [37,38]. The binding of SUV39H1 to HP1–HP1 dimers contributes to its recruitment to nucleosomes [37,39] (Figure 2A, Step 6). In both mice and humans, the suppressor of variegation 3-9 homolog 2 (SUV39H2) has been identified, which has both SET domain and chromodomain, and along with SUV39H1, has H3K9-methyltransferase activity and interacts with HP1α [40,41]. 

SUV39H-mediated establishment of H3K9me3 at PCH requires a pre-modified H3K9me1 substrate [31,40] (Figure 2A, Step 6). Several HMTs are involved in H3K9me1 biosynthesis, such as the SET-containing proteins PRDM3 and PRDM16 (Figure 2B), which belong to the PRDI-BF1 and the *RIZ* homology domain containing (PRDM) family [42], and ESET (Figure 2B), which has been linked to H3K9me1 deposition on PCH during replication, in association with a complex that contains chromatin assembly factor 1 (CAF1) and HP1α [30,43]. 

Interestingly, the combined knockdown of *Prdm3* and *Prdm16* in immortalized mouse embryonic fibroblasts (iMEFs) leads to impairment of MajSat DNA organization, a transition to a more decondensed state, and the upregulation of MajSat RNAs. A similar effect, although to a lesser extent, has been reported upon ESET knockdown in *Suv39h1/h2* double-null iMEFs [32]. These data strengthen the idea that the H3K9 methylated state is crucial for the physiological organization of PCH.

H4K20me3 deposition on PCH is mediated by suppressor of variegation 4-20 homolog 1 (SUV4-20H1) and homolog 2 (SUV4-20H2). These are two SET-containing HMTs that are localized at chromocenters, through their physical interactions with HP1s [25,33] (Figure 2A, Step 2).

Crosstalk between H3K9me3 and H4K20me3 deposition has been described: in *Suv39h1/h2* double-null MEFs, the H4K20me3 and SUV4-20H enzymes show reduced enrichment at PCH compared with wild-type MEFs. On the contrary, in the absence of H4K20me3, H3K9me3 localization is not altered, which suggests that H3K9 trimethylation acts upstream [33,45]. 

A strong decrease in H4K20me3 enrichment on chromocenters, accompanied by reduced accumulation of SUV4-20H2 and HP1γ, has also been described upon the knockdown of the ncRNA *ChRO1* in murine myotubes [21] (see also Section 4).

According to the current model, the presence of H3K9me3 on PCH provides a binding site for HP1s, and through a direct interaction, these recruit SUV4-20H. This SUV4-20H then promotes H4K20 trimethylation in these genomic regions [33,45] (Figure 2A, Step 2).

SUV4-20H1 is dynamically associated with PCH, whereas SUV4-20H2 strongly and stably binds PCH, where it can also act as a structural component [45]. Accordingly, SUV4-20H2 plays a role in nuclear organization and in the dynamics of nuclear pores, whereby it physically interacts with HP1s and binds cohesin subunits [21,45]. It was proposed that SUV4-20H2 mediates PCH compaction by both recruitment of cohesin subunits and shaping of a molecular bridge between HP1s and different PCH regions (Figure 2A, Step 2). Accordingly, *Suv4-20h* double knockout cells have increased chromatin accessibility at pericentric regions and defects in chromocenter organization. Importantly, a large decrease in cohesin subunits at PCH was reported also for *Suv39h1/h2* double-null cells, which thus reinforces the idea of interplay between the H3K9 and H4K20 trimethylation activities [45].

The SUV4-20H enzymes use H4K20me1 as a substrate to produce higher-order methylated forms of H4K20 [60,61] (Figure 2A, Step 2). H4K20 monomethylation is mediated by SET8 (Figure 2B), which is a conserved protein that includes a SET domain and regions that are critical for its methyltransferase activity [58,62]. To date, the exact number of methyl groups added by SUV4-20H to pre-modified H4K20me1 is still debated. The current hypothesis supports the trimethylation activity of SUV4-20H, although several studies have suggested that the structure of the active site of SUV4-20H allows catalysis of only H4K20 dimethylation, which would hypothesize the need for other HMTs for H4K20 trimethylation [59,60,61].

Another hallmark of mammalian PCH in somatic cells is DNA methylation [11]. In mammals, pericentric DNA repeats are predominantly methylated at cytosine 5 of CpG dinucleotides (5meC), and the abundance of this epigenetic mark is characteristic of cell identity. In murine germ cells and preimplantation embryos, MajSat DNA is hypomethylated, whereas in somatic cells, this pericentric DNA is generally hypermethylated [63]. 

In mice, methylation at CpA dinucleotides occurs at MajSat DNA of murine embryonic stem cells (mESCs); conversely, this epigenetic modification has not been seen for the majority of somatic cells [64]. The higher levels of CpA methylation in human ESCs with respect to somatic cells [65] suggest that the function of this epigenetic modification is conserved across species. However, the role of CpA methylation in PCH remains unknown. 

In mammals, methylation of CpG dinucleotides is catalyzed by three DNMTs, each of which plays distinct roles and acts during specific developmental time windows, as well as during specific phases of the cell cycle. During embryogenesis, MajSat DNA is methylated de novo by DNMT3A and DNMT3B [52], with the cooperation of DNMT3L [66,67], which lacks catalytic activity [68]. The methylation pattern established by these de novo enzymes is inherited during DNA replication due to the enzymatic activity of maintenance DNMT1, which is recruited by Ubiquitin-like, containing PHD and RING finger domain 1 (UHRF1) on hemimethylated CpG dinucleotides [69]. Functional crosstalk between the de novo and maintenance DNA methylation machineries has been hypothesized [70]. 

DNA methylation of CpA dinucleotides on PCH of human and murine ESCs appears to correlate with the flanking methylated CpG dinucleotides, and is catalyzed by DNMT3A and DNMT3B, with the contribution of DNMT3L [64,65]. DNMT3A and DNMT3B share common domains, which include an ATRX–DNMT3–DNMT3L (ADD) domain, which mediates the recognition of unmodified H3K4 [71], and a Pro-Trp-Trp-Pro (PWWP) motif, involved in DNA–protein interactions [72] and in targeting at PCH [73]. 

DNA methyltransferase distributions and DNA methylation across the genome are tightly regulated, and require several factors [74]. In particular, DNA methylation of PCH has been linked to the HP1 proteins and H3K9me3 [40]. This thus strengthens the idea that a complex network of interactions takes place in these regions between several factors.

The role of DNA methylation in higher-order PCH organization is still debated. DNA methylation at PCH has a repressive function [9], and its role in the preservation of genome integrity and stability has been investigated through studies of human diseases [9,75,76,77]. Patients affected by types 1 and 2 immunodeficiency, centromeric region instability, facial anomalies (ICF1, ICF2) syndrome show hypomethylation and decondensation of pericentric satellites II and III [9,78]. It has been proposed that, in ICF syndrome, decreased DNA methylation of these satellites causes their decondensation, which leads to an accumulation of unresolved intermediates during homologous recombination, with the consequent chromosomal rearrangements [75,77]. In support of this hypothesis, Volpi and coworkers [79] reported a correlation between ICF-related satellite hypomethylation, PCH decondensation, and the subsequent alterations to heterochromatin organization [79], and these alterations may affect the maintenance of the silencing of specific loci [23,80]. 

PCH hypomethylation has also been correlated to cell senescence [81,82] and cancers [83,84]. In mammals, CpG methylation generally represses gene expression, although the role of DNA methylation for the prevention of the spurious expression of transcripts derived from satellite sequences is not completely clear. Satellite transcript expression is not affected in *Dnmt1*-null and *Dnmt3a*/*Dnmt3b* double-null mESCs, in which DNA methylation is not completely abrogated; however, in *Suv39h1/h2* double-null mESCs, in which both histone methylation and DNA methylation at PCH are reduced, there is modest upregulation of MajSat expression. This suggests a synergistic effect of DNA and histone methylation in transcriptional repression of satellite repeats [40]. A role for DNA methylation in silencing satellite DNA expression has also been demonstrated in cancer cells, which is characterized by the hypomethylation of pericentric DNA repeats [83,84] and altered pericentric transcript expression [8,85]. 

A function of pericentric DNA methylation in the inhibition of binding of polycomb group (PcGs) proteins to PCH has been proposed. In support of this, the induced demethylation of MajSat repeats in mESCs triggers the recruitment of polycomb repressive complex (PRC) 1 and 2 to these regions [86]. PRC accumulation at pericentric regions has also been described in cancer cells, which are characterized by extensive DNA hypomethylation [87]. Interestingly, this nonphysiological accumulation of PcGs on PCH appears to reduce their binding to canonical sites, which might result in alterations to gene expression [86]. 

Correlations between histone methylation and DNA methylation at PCH have been reported. Peters and coworkers [40] showed that the combined depletion of *Suv39h1/h2* in mESCs leads to large delocalization of DNMT3B from PCH, which parallels the significant impairment of DNA methylation in these regions, as well as the slight upregulation of MajSat transcripts [40]. DNMT3B physically interacts with HP1α and HP1β, which then localize to PCH by binding SUV39H-produced H3K9me3 [40,88] (Figure 2A, Steps 5 and 7). These findings support the hypothesis that SUV39H-mediated H3K9me3 production provides a binding platform for HP1s, which can then recruit DNMT3B, which is responsible for DNA methylation at PCH (Figure 2A, Steps 5 and 7). Moreover, a physical interaction between DNMT3A and DNMT3B has been described, as well as a binding of DNMT3A to HP1s [16,40] (Figure 2A, Steps 5 and 7).

The methylation status of MajSat repeats then regulates the methylation level of histone tails at PCH, such as H3K27. In *Suv39h1/h2* double-null mESCs, which show hypomethylation of MajSat DNA, H3K27me1 is lost from the chromocenters, which are instead enriched in H3K27me3 and monoubiquitinated lysine 119 on the H2A histone (H2AK119ub1) [31,86]. In *Dnmt1*/*Dnmt3a*/*Dnmt3b* triple knockout (*Dnmt* TKO) mESCs, which show depletion of DNA methylation, PRC1 is recruited to PCH [89] and mediates deposition of H2AK119ub1, which then leads to the binding of PRC2, with the consequent formation of H3K27me3 [86]. Moreover, *Dnmt* TKO mESCs and human cells that lack DNMT1 or DNMT3B show reduced accumulation of H3K9me3 at pericentric regions [9,10,90]. Taken together, these findings underline the tight crosstalk between DNA methylation and deposition of H3K9me3 and H3K27me1 on PCH.

## 3. Readers of Epigenetic Modifications that Control Pericentric Heterochromatin Status 

H3K9me3 and H4K20me3 histone marks represent anchors for the attachment of HP1s to PCH [44,91], with HP1s thus highly enriched in these heterochromatic regions [20]. HP1s belong to a family of highly conserved proteins [92] that in mammals includes three isoforms, HP1α, HP1β, and HP1γ [93], which show different genomic distributions that include both euchromatic loci [94,95] and heterochromatic regions [96]. HP1s interact with several molecular partners and can undergo post-translational modifications [16,97]. *Hp1α*, *Hp1β*, and *Hp1γ* knockout mice show different phenotypes, which rules out their functional redundancy [98,99,100,101]. HP1s contain a chromodomain that is responsible for their anchoring to H3K9me2/me3 on PCH, and a chromo-shadow domain that mediates HP1 dimerization and interactions with other partners [44,95,102,103]. 

Specific post-translational modifications have been shown to regulate the localization of HP1s at chromocenters. In mice, SUMOylated HP1α binds the forward transcript derived from MajSat repeats (*MajSat-fw*), which mediates its de novo targeting to PCH [54]. In contrast, retention of HP1α in these regions requires a deSUMOylation event [104].

HP1s bind H3K9me3 at PCH to recruit SUV39H enzymes through direct interactions, and to mediate the spread of H3K9 trimethylation onto adjacent nucleosomes (Figure 2A, Step 6). This then provides a scaffold for the binding of additional HP1 molecules. This mechanism has been defined as a “self-sustaining loop” [16,44,105], and it is also involved in the propagation of H4K20me3 deposition across PCH [25,45,91]. Recently, the binding of HP1α with CCCTC-binding factor (CTCF) has been described, and it was proposed to regulate higher-order PCH organization through interplay with some of the typical histone modifications of these regions [91].

Highly methylated MajSat DNA repeats provide a binding platform for proteins that belong to the methyl-binding domain (MBD) family, a particular group of “readers” of epigenetic marks that establish a functional link between DNA methylation and histone modifications [50]. MeCP2 is one of these factors, and it is an epigenetic modulator of chromatin architecture that is strongly enriched at PCH [20,106] (Figure 2A, Steps 4 and 9). It is mutated in Rett syndrome, a severe neurological disorder [107]. MeCP2 is a master regulator of gene expression that can mediate both transcriptional activation and repression, depending on its interactions with its different molecular partners. Moreover, MeCP2 is involved in protection of the genome from aberrant histone acetylation, and in modulation of histone H1 genomic density (reviewed in [14,108]). MeCP2 is specifically accumulated at genomic regions enriched in methylated CpGs [109], although there is evidence that suggests that MeCP2 also binds methylated cytosines in a nonCpG context [110], as well as nonmethylated DNA [111,112]. 

It was recently demonstrated that in neurons the fraction of MeCP2 that is stably bound to chromatin is higher in comparison with the distribution of other transcription factors [113]. MeCP2 behavior in this context depends on both the integrity of its MBD and the DNA methylation [113]. Indeed, several mutations in the MBD of MeCP2 that can cause Rett syndrome, such as R106W, result in reduced binding of MeCP2 to methylated DNA [112,114], and an increased rate of MeCP2 diffusion in the nucleus [113]. MeCP2 has also a transcriptional repression domain (TRD) and a C-terminal region. These are involved in transcriptional silencing through recruitment of several molecular partners, which include histone deacetylases (reviewed in [14]) (Figure 2A, Step 9). 

**Figure 3:**
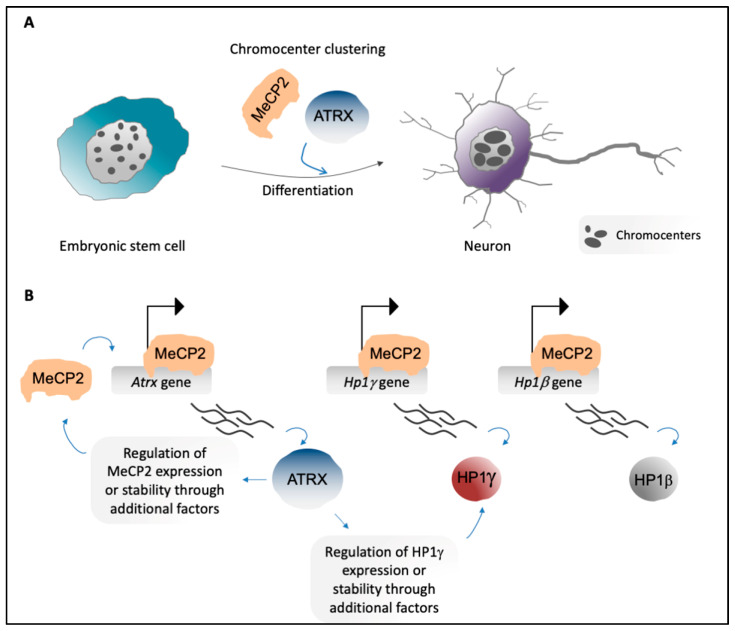
(**A**) Embryonic stem cells show high numbers of chromocenters per nucleus (left). In neurons, chromocenters increase in size and decrease in number due to aggregation of PCH of different chromosomes (chromocenter clustering) [18,20] (right). MeCP2 [18] and ATRX [20] are important players in chromocenter clustering during neural differentiation. (**B**) MeCP2 directly promotes expression of genes that encode PCH-associated factors, including *Atrx*, *Hp1γ*, and *Hp1β* (top). ATRX regulates expression and/or stability of MeCP2 and HP1γ, probably through the involvement of additional factors [20] (bottom).

The role of MeCP2 as a key player in the reorganization of PCH is now well established. During myogenic [19] and neural [18] differentiation, the chromocenters undergo wide spatial reorganization (Figure 3A), which is accompanied by increased MeCP2 levels. MeCP2 ectopic expression in murine myoblasts is sufficient to induce aggregation of pericentric regions [19], and along the same lines, neurons lacking MeCP2 show defective chromocenter clustering [18]. Moreover, MeCP2 physically interacts with HP1s and contributes to their recruitment to PCH [57] (Figure 2A, Steps 4 and 9). Several Rett-syndrome-causing mutations in MeCP2 MBD also affect the localization of MeCP2 on PCH and/or the induction of chromocenter aggregation [115,116,117]. 

MeCP2 can undergo several post-translational modifications (reviewed in [118]). Among these, poly(ADP-ribosyl)ation has been hypothesized to modulate MeCP2 affinity for chromatin, as well as its induction of PCH condensation [119]. Moreover, MeCP2 phosphorylation on serine 80 and serine 229 regulates MeCP2 binding with several of its partners, including HP1s [120].

Of note, we have recently proposed that, in neurons obtained by in vitro differentiation of mESCs, MeCP2 directly regulates the expression of *Hp1β* and *Hp1γ* [20] (Figure 3B), thus strengthening the idea that the two roles of MeCP2 as a chromatin organizer and a transcriptional modulator are tightly interconnected. Moreover, an interaction between MeCP2 and MBD2, another member of the MBD family, has been described (Figure 2A, Step 4). MBD2 has been hypothesized to contribute to the global reorganization of pericentric regions [49]; as seen for MeCP2, MBD2 accumulates on PCH and shows increased levels during myogenic differentiation, and its ectopic expression in myoblasts induces aggregation of chromocenters [19,121]. 

MeCP2 has been hypothesized to modulate DNMT3A-dependent DNA methylation by acting as both an activator and a repressor of this DNMT, depending on genomic localization. Indeed, MeCP2 recruits DNMT3A to specific regions of the genome, including PCH. The methylated DNA can then provide a binding platform for MeCP2, thus defining a potential positive-feedback mechanism that contributes to the maintenance of a stable DNA methylation state (Figure 2A, Step 4). On the other hand, DNMT3A catalytic activity is inhibited by the interaction with MeCP2, although it can be restored by unmodified H3K4, which is enriched in PCH. Accordingly, MeCP2 has been proposed to inhibit the activity of DNMT3A in regions that are enriched in H3K4 permissive histone modifications, which are poorly represented for PCH, to protect the genome from aberrant DNA methylation [51].

MeCP2 co-localizes at chromocenters with ATRX [20], which is a nuclear epigenetic factor that belongs to the switch/sucrose non-fermentable (SWI-SNF) protein family [122]. SWI-SNF proteins play roles in several biological processes, such as DNA recombination and repair, transcriptional regulation, and remodeling of nucleosomes [123]. ATRX is mutated in the complex X-linked disorder known as ATR-X syndrome [124,125], and similar to MeCP2, ATRX can act as both a transcriptional regulator and an organizer of higher-order chromatin structure [126]. Interestingly, ATRX is involved in MeCP2-mediated chromocenter clustering during neural and myogenic differentiation [20,21] (Figure 3A). 

We recently dissected the interplay between ATRX and MeCP2 through the demonstration that, in mESC-derived neurons, MeCP2 directly promotes *Atrx* expression. ATRX also positively regulates MeCP2 expression, potentially through an indirect mechanism [20] (Figure 3B). MeCP2 physically interacts with ATRX [55,56] (Figure 2A, Steps 8 and 9), and this interaction is required for localization of ATRX to PCH in neurons [20,56]. Of note, the R270X and G273X Rett-syndrome-causing mutations of MeCP2 lead to decreased accumulation of ATRX at PCH, although MeCP2 enrichment at chromocenters and its affinity for ATRX are not lost. This suggests that in neurons, MeCP2 promotes the formation of particular PCH conformations that can then provide binding sites for ATRX [55]. In addition, we have highlighted that ATRX contributes to MeCP2 enrichment at PCH in neurons [20].

ATRX contains an SNF2 homology domain that mediates the remodeling of nucleosomes through a DNA-dependent ATPase activity [127], along with an ADD domain that is involved in ATRX localization to chromatin, including PCH. ATRX binds PCH that is enriched in both unmodified H3K4 and H3K9me3 [46,47] (Figure 2A, Steps 3 and 8). Moreover, HP1s can provide an additional binding site for ATRX [128] (Figure 2A, Steps 3 and 8). Mutations in the ATRX ADD domain and the HP1-interaction motif affect the binding of ATRX to chromocenters [46,47]. ATRX also contributes to the accumulation of HP1α and HP1γ at PCH, and regulates the expression of HP1γ in mESC-derived neurons [20] (Figure 3B).

ATRX physically interacts with the histone chaperone Fas death domain-associated protein (DAXX), which can then bind to the H3.3 histone variant (Figure 2A, Step 3). The ATRX/DAXX complex has nucleosome remodeling activity and mediates replication-independent deposition of H3.3 on several heterochromatic regions, including PCH (Figure 2A, Step 3) [21,48,126]. The meaning of this H3.3 deposition across pericentric regions has not yet been completely defined. It has recently been shown that knockdown of ATRX, DAXX, or H3.3 leads to impaired chromocenter clustering during myogenic differentiation [21], with a role for the muscle-specific transcript *ChRO1* in DAXX enrichment at PCH highlighted. Knockdown of *ChRO1* leads to reduced accumulation of the DAXX/H3.3 complex at chromocenters, which is accompanied by decreased enrichment of RNA polymerase II. The authors hypothesized that H3.3 deposition on pericentric regions can activate transcription of MajSat RNAs that then play a role in chromocenter clustering [21]. These data are in agreement with the previously observed downregulation of MajSat transcripts in DAXX-null MEFs and upon ATRX or H3.3 knockdown [48].

## 4. Role of Non-Coding RNAs in Pericentric Heterochromatin Organization 

Non-coding RNAs are considered to be hallmarks of human and mouse PCH, and their recruitment to pericentric DNA represents a critical step for PCH organization and maintenance [20,21,53,54,129,130,131]. Many reports have highlighted that the accumulation of several PCH-related proteins at chromocenters is dependent on an RNA moiety (Figure 2A, Steps 6 and 8), although the global spatial organization of PCH is not affected by ablation of the RNA component [20,129,130,131]. 

For about 20 years, it has been known that satellite DNA repeats included in PCH are actively transcribed despite the heterochromatic nature of PCH [40,132], which is supported by the presence of several transcription factors at pericentric DNA [133]. Under physiological conditions, pericentric satellite DNA expression is low, and is temporally and spatially regulated [11,134,135]. However, expression of satellite RNAs has been reported in several biological contexts and under different conditions [136]. 

In mice, pericentric satellite DNA is primarily transcribed by RNA polymerase II, which produces molecules of heterogeneous lengths [40,136,137] from both the forward and reverse strands [40] (*Majsat-fw*, *Majsat-rv*, respectively). Conversely, RNA polymerase I is the main enzyme for transcription of pericentric satellite DNA in humans [130]. The increasing literature on this underlines that transcripts that originate from pericentric satellite DNA are not the result of transcriptional noise, as previously believed; instead, they have specific biological functions. However, the biological relevance of the single-stranded or double-stranded forms of these ncRNAs in mammals is still debated [53,54,134,138,139]. 

In mice, distinct roles for the *MajSat-fw* and *MajSat-rv* RNAs have been postulated. MajSat RNAs are involved in chromocenter formation and the maintenance of higher-order PCH structures [4,90,134]. During the first cleavage stages of murine embryogenesis, PCH undergoes rapid reorganization, and MajSat transcripts play a critical role in this. At the two-cell stage, when the ring structures progressively reorganize to form chromocenters, there is a burst in transcription of MajSat RNAs that is both spatially and temporally regulated [135]. During the cell cycle, the transcription of *MajSat-fw* occurs during S phase, and increases up to S/G2 phase, when upregulation of *MajSat-rv* occurs. At the four-cell stage, when chromocenters are completely formed, transcription of both *MajSat-fw* and *MajSat-rv* is strongly downregulated [135]. Remarkably, knockdown of MajSat RNAs at the two-cell stage results in developmental arrest before the completion of chromocenter formation [135]. Subsequently, *MajSat-rv* was identified as the transcript that is required for chromocenter formation at the two-cell stage [134]. Fine-tuned regulation of MajSat transcript expression has also been reported in various differentiation model systems [140,141], and increased levels of MajSat RNAs have been reported during neuronal differentiation [142]. The mechanism that controls MajSat RNA expression is still debated, although it appears to be dependent on the methylation status of histones [40]. Similarly, Tapscott and coworkers [143] demonstrated that the bidirectional transcription of satellite II RNAs is also temporally regulated in human early embryogenesis, and that this process is primarily regulated by double homeobox 4 (DUX4). In mice, MajSat transcripts accumulate at chromocenters [21,54,129], and can form DNA:RNA hybrids [53]. In humans, only the pericentric α-satellite RNA associates in cis with PCH of mitotic chromosomes, and thus not the β-satellite and satellite III transcripts [130]. 

During murine myogenic differentiation, MajSat RNAs have been reported to play roles in chromocenter clustering [21]. However, whether these transcripts are directly involved in this process remains unclear. Accordingly, one of the well-characterized functions of pericentric satellite transcripts is their involvement in higher-order PCH organization, through the tethering and anchoring of PCH-related proteins to chromocenters [53,54,129,130]. In the murine context, SUV39H1 and SUV39H2 association to PCH is dependent on MajSat transcripts [53], and the *MajSat-fw* RNA tethers small ubiquitin-like modifier (SUMO)-modified HP1α at chromocenters [54]. Furthermore, the interaction of MajSat transcripts with scaffold attachment factor B (SAFB) is important to stabilize PCH [129], which strengthens the hypothesis that transcripts derived from pericentric DNA repeats are involved in higher-order PCH organization. Altogether, these findings indicate that MajSat transcripts serve as a scaffold for the formation of multiprotein complexes at PCH.

As well as MajSat transcripts, other ncRNAs can modulate PCH architecture. The muscle-specific ncRNA *ChRO1* is enriched at chromocenters in murine myotubes, and its contribution to higher-order PCH organization has been shown [21]. *ChRO1* is required for the targeting of SUV4-20H2, HP1, MeCP2, and cohesin subunits to chromocenters, a process that then promotes correct DNA methylation and H4K20me3 deposition. Furthermore, *ChRO1* plays a role in chromocenter clustering during myogenic differentiation, through the promotion of deposition of H3.3, and thus MajSat RNA expression [21]. 

**Figure 4:**
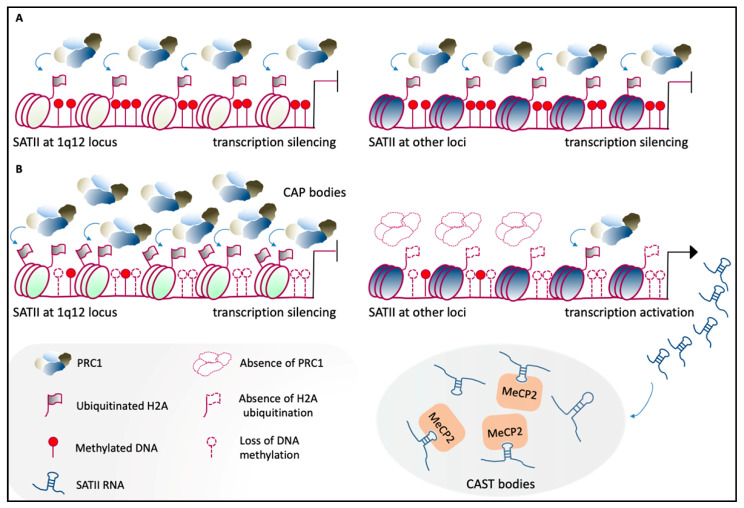
Human PCH organization in health and disease. (**A**) Under normal physiological conditions, satellite II (SATII) at the 1q12 locus (left) and at other chromosomal loci (right) is highly methylated at the DNA level and binds the PRC1 complex, which mediates H2A ubiquitination. This molecular landscape leads to the maintenance of the transcriptionally inactive state [151]. (**B**) In cancer cells, the loss of methylation across SATII loci causes hyper-accumulation of PRC1 proteins at SATII at the 1q12 locus, which leads to the formation of cancer-associated polycomb (CAP) bodies (top-left). This mechanism maintains the silencing of 1q12-SATII, which is reinforced by increased H2A ubiquitination. At other loci, SATII shows less accumulation of ubiquitinated H2A, and becomes transcriptionally active (top right). This leads to the formation of cancer-associated satellite transcript (CAST) bodies, in which there is an aberrant accumulation of SATII RNAs. These aggregates sequester epigenetic factors, including MeCP2. Adapted from [151].

Aberrant expression of mammalian satellite transcripts in response to cell stress and senescence, and in diseases, including cancers, has been widely demonstrated [144,145] (see also below). In human cells, heat shock promotes expression and accumulation of transcription factor heat-shock factor 1 (HSF1) and RNA polymerase II at nuclear stress bodies (nSBs), which then induce the expression of satellite III of chromosome 9 [146,147]. Additionally, DAXX-mediated upregulation of satellite III RNAs upon heat shock has been shown [148]. However, the role of satellite III RNAs in response to stress is still debated. On the one hand, satellite III transcripts are associated to nSBs and appear to be involved in self-organization of these structures [149]; on the other hand, satellite III RNAs that accumulate under stress conditions have been proposed to act as “sponges,” and thus to sequester factors involved in transcription and splicing, with subsequent broad transcriptional downregulation [150]. Likewise, similar mechanisms have been characterized recently in cancers, where aberrant expression of pericentric RNAs occurs in both humans and mice [144,151]. Studies by Lawrence and coworkers [151] support the hypothesis that human satellite II DNA and RNA act as molecular sponges to modulate the availability of epigenetic factors, whereby these are sequestered under pathological conditions. Indeed, cancer-related DNA demethylation of satellite II at 1q12 is responsible for the recruitment of PRC1 to cancer-associated polycomb (CAP) bodies, and this leads to derepression of other satellite II DNA loci. On the other hand, aberrantly expressed human satellite II RNA sequesters MeCP2 into cancer-associated satellite transcript (CAST) bodies, which results in the reduced availability of this epigenetic regulator [151] (Figure 4). Of note, the role of satellite II transcripts as molecular sponges was also proposed by Tapscott and coworkers [143]. 

## 5. Functional Roles of Pericentric Heterochromatin 

At present, the biological functions of PCH are poorly understood and are still under discussion. Studies performed over recent decades using several eukaryotic model organisms have highlighted that PCH is involved in multiple processes that are crucial for safeguarding the cell, such as the maintenance of the boundary between euchromatin and the centromere core [152], and for centromere function [153]. Moreover, PCH is important for correct sister chromatid cohesion and chromosome segregation during mitosis [154], as processes mediated by cohesins [155], and for the suppression of centromeric recombination during meiosis [156]. PCH also plays important roles in the preservation of genome integrity. Indeed, specific post-translational histone modifications and proteins associated with PCH (e.g., cohesins) are crucial for the maintenance of repeat stability, through the suppression of homologous recombination, for the control of the three-dimensional organization of damaged repeats, and for the reduction of aberrant recombination [157]. In line with this, the loss of PCH structure and function gives rise to genome instability through incorrect recombination between heterochromatic repeats [158]. PCH is also implicated in the maintenance of transcriptional silencing of satellite DNA [133], the aberrant overexpression of which, in mammals, has been associated with defects in centromere structure and the mitotic spindle, and with aberrant chromosome segregation during mitosis [159].

An intriguing function of PCH is related to the organization of silent nuclear compartments, which spatially are located away from the actively transcribed genome. For many years, it was postulated that these structures form a repressive environment that is highly enriched in silencing factors [4,22], and where transcriptionally inactive genes are located, to establish and/or maintain a differentiated state [23,160,161]. Several studies over the last two decades have supported this idea. Using cycling and noncycling murine lymphocytes, it was demonstrated that genomic loci are spatially associated with PCH only in cell types where these loci are silenced, which thus suggests that the nuclear spatial organization of these genes depends on their transcriptional state [160]. Similar findings have also been reported in the human context. Indeed, analysis of the human β-globin locus sustain the hypothesis of a sequential model of gene activation during erythroid commitment that involves first the relocation of the locus away from PCH, then the local hyperacetylation of histone H3, and finally the transcriptional activation of the β-globin locus [80,162,163]. Furthermore, during human myogenic differentiation, the irreversible silencing of E2F target genes that are permanently silenced in terminally differentiated myogenic cells is mediated by their repositioning close to PCH, and their enrichment in trimethylated H3K9. On the other hand, transient repression of the same E2F target genes in quiescent and early-G1 cells, is independent of the proximity of the target genes to PCH, which supports the existence of two different repressive mechanisms under these two biological conditions [164]. However, the association between PCH proximity and transcriptional repression is still debated [165,166,167,168,169].

More recently, a correlation between transcriptional activity and spatial association with PCH was investigated through a genome-wide strategy in mouse cells [23]. This study identified ∼1000 pericentromere-associated domains (PADs), where their genomic distribution is alternated with non-PADs. The authors highlighted that PADs are gene-poor chromosomal regions that contain loci with low expression levels and are enriched in repressive histone modifications, such as methylated H3K9 and H4K20, which show high levels of DNA methylation and are devoid of permissive histone modifications, as is methylated H3K4. Moreover, this study showed that the induction of forced proximity of an actively transcribed locus to chromocenters is sufficient to promote silencing of the locus. This evidence strongly supports the hypothesis that PCH plays a crucial role in the formation of repressive nuclear compartments and the resulting silencing of the associated genes [23].

## 6. Pericentric Heterochromatin in Human Diseases

In the previous sections, we illustrated the structure and function of this very particular part of the genome, PCH, and described its interactions with molecules of various natures and its epigenetic signature. Here, we will link genetic mutations that cause diseases, including cancers, with perturbations of PCH, which might contribute to the clinical manifestation of these pathologies. Additionally, we will describe the versatility of PCH-based methods to improve gene therapy strategies. 

### 6.1. Genome Alterations in ICF Syndrome Type 1

Type I immunodeficiency, centromeric region instability, facial anomalies (ICF1) syndrome (OMIM, 242860) is a puzzling disease that fits into the scenario of PCH-related defects. The ICF1 pathology is a chromatin disease [170,171,172] that manifests as a rare autosomal-recessive disorder with severe immunodeficiency, craniofacial anomalies, and chromosome instability [173]. ICF1 is caused by mutations in DNMT3B [174], which is responsible for the hypomethylation of satellites II and III, which is considered the hallmark of ICF syndrome. However, hypomethylation of other genomic regions has been reported, such as genes belonging to pseudoautosomal region 2 (PAR2) of the X chromosome [170,171,172]. On this basis, *PAR2* gene hypomethylation in the nucleus of cells from patients with ICF1 has been associated with altered three-dimensional positioning of PAR2 genes with respect to the chromosome territories [170]. It has also been proposed that significant hypomethylation of satellites II and III at chromosomes 1, 16, and sometimes 9, is responsible for the altered organization of PCH and the chromosomal instability observed in ICF1 [9,75,77].

### 6.2. Lamin A Alterations in Laminopathies and Mutation Effects on Genome Architecture

Laminopathies are pathologies that are caused by mutations in the *LMNA* gene that encodes lamin A/C, or in genes that encode lamin-binding proteins. Lamins act as structural scaffolds that maintain nuclear integrity, anchor heterochromatin at the nuclear periphery, and regulate gene expression [175]. The majority of laminopathies have been linked to A-type lamin, and these include myopathies, lipodystrophies, and ageing syndromes, among others [175]. Laminopathies are often associated with severe alterations to the nuclear and heterochromatin architectures [175].

The autosomal dominant and the X-linked forms of Emery–Dreifuss muscular dystrophy (AD-EDMD; OMIM, 181350; XL-EDMD; OMIM, 310300; respectively) are two myopathies that share similar clinical symptoms, which include skeletal muscle and cardiac muscle defects. AD-EDMD is caused by mutations in the *LMNA* gene, whereas patients with XL-EDMD show mutations in the *EMD* gene, which encodes emerin, a protein associated with the inner nuclear membrane [175]. Both of these disorders are characterized by heterochromatin and nuclear defects, which include heterochromatin loss or detachment from the nuclear periphery [175]. Moreover, the lamin A R453W mutation that causes AD-EDMD induces delocalization of H3K9me3 from PCH [176]. It has been proposed that mutations in *LMNA* and *EMD* are responsible for an altered transcriptional program that results in impaired myogenic differentiation [177].

Mandibuloacral dysplasia (MADA; OMIM, 248370) is a laminopathy that is characterized by lipodistrophy, skeletal abnormalities, metabolic alterations, and postnatal growth retardation [178], and it is due to mutations in *LMNA* that are responsible for the accumulation of the lamin A precursor [178]. Cells from patients with MADA show invaginations in the nuclear envelope, a loss of peripheral heterochromatin, and delocalization of HP1β, H3K9me3, and their partner lamin B receptor. Of note, these defects are pronounced in older MADA cells [178]. 

Another relevant class of laminopathies associated with heterochromatin defects is represented by premature aging disorders, which include Hutchinson–Gilford progeria syndrome (HGPS) (OMIM, 176670). Patients with HGPS have an aged appearance, and show growth retardation, bone deformations, and cardiovascular problems [179]. HGPS is caused by mutations in *LMNA*, the most common of which results in the expression of a truncated form of lamin A (progerin). This accumulates in the nucleus with age-dependent deleterious effects on chromatin structure and transcription [179]. As reported for other laminopathies, fibroblasts from patients with HGPS show significant changes in nuclear shape and almost a complete loss of heterochromatin from the nuclear periphery [179]. Moreover, these fibroblasts show increased accumulation of H4K20me3, reduced accumulation of H3K9me3, and impaired interactions between H3K9me3 and HP1α at the nuclear periphery [179]. The 433G>A HGPS-causing mutation (E145K) lies in the central rod domain of lamin A that is involved in polymerization of the nuclear lamins. This thus results in profound defects in nuclear architecture, including alterations to PCH, abnormally clustered centromeres, and mislocalized telomeres [180]. These findings underline the central role of the lamin A rod domain for global chromatin organization [180].

Similar defects in PCH epigenetic marks observed in patients with HGPS have also been shown in cells from healthy aged individuals, in comparison with those of healthy young subjects. This allows for the hypothesis that lamin A–mediated epigenetic alterations are also implicated in physiological ageing [179]. Indeed, such progeric laminopathies represent ideal models for the study of normal ageing processes.

In the last decade, increasing genome-wide studies have highlighted a significant impact of *LMNA* mutations on the organization of lamina-associated domains (LADs), which are portions of heterochromatin that are enriched in H3K9me3 and are connected with the nuclear envelope [181]. As an example, Hoffman and coworkers demonstrated that the R453W mutation that causes AD-EDMD significantly increases the number of LADs. Several other laminopathy-causing *LMNA* mutations also impair the correct configuration of LADs, although to different extents [181]. 

Overall, these findings suggest that disease-related lamin A mutations can drastically impact to a global level on interactions between lamin A and PCH. This results in the impaired localization of PCH at the nuclear periphery, which thus impacts on gene expression programs.

### 6.3. Pericentric Heterochromatin and Cancer

A loss of DNA methylation of satellite II of chromosomes 1 and 16 has been reported for many types of cancers [182,183], which has indicated the importance of correct PCH methylation in carcinogenesis. DNA hypomethylation can occur very early in tumorigenesis, and it has been strongly linked with tumor progression [183]. Accordingly, in ovarian carcinoma, hypomethylation of satellite II of chromosome 1 has been associated with tumor grade, and identified as a marker of risk of relapse [184]. However, the role of satellite DNA hypomethylation in cancers is not fully understood to date. Satellite methylation might be involved in genome stability and correct chromosomal segregation, as previously postulated [185]. Moreover, rearrangements of the pericentric satellite II of chromosome 1 are frequent events in hematological malignancies, such as non-Hodgkin’s lymphoma and multiple myeloma, and they have been reported to perturb the nuclear organization of PCH of chromosome 1 [186,187]. 

Overexpression of pericentric satellite repeats has also been reported for many types of cancer [85,136], and this might arise from a loss of DNA methylation at these regions [188]. However, whether this satellite overexpression is associated with genome stability is still debated [136]. Both DNA and RNA of satellite II appear to sequester chromatin-regulatory proteins, such as MeCP2 and PRC1, within cancer-specific nuclear bodies, by impacting on their distribution, which might have significance in cancer-related alterations to the genomic architecture [151]. The mislocalization of chromatin-associated proteins within the nuclear structure triggered by demethylation of the so-called “junk” repeats indicates that they might contribute to the altered epigenomic landscape in cancers. 

### 6.4. Lentiviral Integration in PCH as a Strategy for Gene Therapy

Commonly used gene therapy strategies using lentivirus infection involve preferential integration of a lentivirus to active transcription units, a process that is mediated by the host protein lens epithelium-derived growth factor (LEDGF/p75), which interacts with the lentiviral integrases [189]. However, random lentivirus integration might interrupt essential genes or activate proto-oncogenes, which might have disastrous consequences. To overcome this problem, Debyser and coworkers [190] exchanged the LEDGF/p75 chromatin-interaction binding domain with HP1β, a factor that binds H3K9me2 and H3K9me3, which are histone modifications that are enriched in PCH. This strategy allowed them to target viral integration outside of the gene-rich genome and thus in regions that are characterized by a repressive state, such as PCH. Of note, the integration of the chimeric vectors in these silent compartments did not affect expression of the chimeric lentiviral vector, which remained efficient. This study establishes a PCH-based strategy to control the integration site selection of lentiviral vectors [190].

## 7. Conclusions

Pericentric heterochromatin is a highly organized structure that arises from the orchestrated actions of several specialized factors. Among these, multiple enzymes “write” the molecular signature of PCH by establishing repressive epigenetic marks that include DNA and histone methylation, which serve as docking sites for particular “readers” and structural components. These factors can act as anchors for the attachment of enzymes that mediate the establishment of the aforementioned epigenetic modifications, with a contribution from ncRNAs. This mechanism is based on a tight interplay between DNA methylation, histone modifications, ncRNAs, and the “readers” and “writers,” and it gives rise to positive feedback-loop that ensures the establishment and maintenance of a stable repressive state, as well as its inheritance during the cell cycle. Alterations of some of these elements can lead to the destabilization of the PCH structure, which strengthens the idea of a tightly organized compartment.

It is worth noting that each factor that is related to PCH plays several roles. SUV4-20H2, for instance, can function both as the enzyme responsible for trimethylation of H4K20 and as a structural component that mediates chromatin compaction through the binding of additional factors. Similarly, both MeCP2 and ATRX can modulate the expression of some PCH-associated proteins and contribute to their localization at chromocenters.

To date, the biological functions of PCH have not been completely defined. It has been proposed to play roles in several physiological processes, such as chromosome segregation, the preservation of genome stability, and the formation of silent compartments where genes are repressed in trans. Alterations of PCH architecture have been shown in different classes of diseases, including ICF syndrome, laminopathies, and cancers. However, further studies are required to shed light on the biological significance of this repressive compartment, and on its correlation with human diseases.
